# Analysis of Sedation Accident Records Available in the Japan Council for Quality Health Care Public Database

**DOI:** 10.7759/cureus.54793

**Published:** 2024-02-23

**Authors:** Uno Imaizumi, Hidetaka Kuroda, Shota Tsukimoto, Norika Katagiri, Takuro Sanuki

**Affiliations:** 1 Department of Dental Anesthesiology, Kanagawa Dental University, Yokosuka, JPN

**Keywords:** p-mshell model, respiratory depression, monitoring, medical accident, sedation

## Abstract

Objective: Medical accidents occur frequently. However, only a few studies have been conducted on sedation-related medical accidents. This study aimed to classify sedation accidents and analyze their causes using the (Patient-management Software Hardware Environment Livewear (P-mSHELL) model.

Methods: The Japan Council for Quality Health Care database on medical accidents was used. Sedation accidents that occurred during procedures between January 2010 and June 2022 were included. After examining the accident details for several variables, the accident factors were classified by factors in the P-mSHELL model, and statistical analyses, including multivariate analysis of each factor and outcome (presence or absence of residual disability), were conducted.

Results: Regarding the influence of the P-mSHELL factors on outcome, P factor (odds ratio = 6.347, 95% confidence interval = 2.000, 20.144) was a factor for having disability. There was a significant association between outcome and accident timing (that is, preoperative, intraoperative, or postoperative; *p *=0.01). No significant association was found between the outcomes and types of drugs used (*p *=1, 0.722, 0.594); however, there was a significant association between the incidence of respiratory depression and multiple drug use (*p *<0.001).

Conclusions: To prevent sedation accidents, it is important to monitor patients throughout the perioperative period. However, it is especially important to know the patient's condition in advance, and strict postoperative management is essential, especially for high-risk patients, to prevent serious accidents.

## Introduction

Sedation is widely practiced in the medical and dental fields. In dentistry, sedation is often used for dental procedures in patients with dental phobia and minor surgery. In medicine, sedation is used in intensive care units, palliative care, and for many endoscopic procedures, surgeries, and resections. Many guidelines have been published on sedation. In 2018, the American Society of Anesthesiologists (ASA), the American Society of Oral and Maxillofacial Surgeons, the American College of Radiology, and others jointly published new guidelines for "Moderate Sedation During Procedures" (Practice Guidelines for Moderate Procedural Sedation and Analgesia 2018: ASA-SED2018). These guidelines focus specifically on moderate sedation during procedures, with particular emphasis on (1) pre-sedation evaluation, (2) monitoring, (3) sedation personnel and emergency response systems, and (4) recovery care [1].

In Japan, there is a "Practical Guide for Safe Sedation" by the Japanese Society of Anesthesiology, "Guidelines for Sedation in Endoscopic Procedures" by the Japanese Society of Gastrointestinal Endoscopy, and "Guidelines for Intravenous Sedation in Dental Care" and "Practical Guide for Deep Sedation in Dental Care" by the Japanese Society of Dental Anesthesiology. Despite the development of these various guidelines to safely administer sedation and prevent medical accidents, medical accidents have repeatedly occurred. However, few studies have analyzed medical accidents due to sedation [2]. Understanding of the factors and characteristics of accidents by those who perform sedation should help prevent new accidents. The purpose of this study was to analyze sedation-related accident reports and understand the factors and characteristics of accidents and help improve the safety of sedation in the future. In this study, medical accidents during sedation in Japan were analyzed for their causes using the P-mSHELL model (Patient-management Software Hardware Environment Liveware model), which is used in medical accident analysis, based on a secondary analysis of data reported by the Japan Council for Quality Health Care. To our knowledge, this is the first study to investigate the causes of sedation-related medical accidents based on accident reports from medical institutions throughout Japan. The P-mSHELL model is an analytical model specialized for medical accident analysis that adds patient and management factors to the SHEL model, which has been used in aviation accident analysis [3,4] and applied to medical accident analysis [5,6] considering the special characteristics of medical care.

## Materials and methods

This study was conducted using data from January 2010 to June 2022 from the Japan Council for Quality Health Care. This data is open-access data and is available to the public on the web. Since confidential information had already been removed and all information was anonymized and published in such a way that individuals and institutions could not be identified, approval from an institutional ethics board was not required.

The purpose of the Project to Collect Medical Near-Miss/Adverse Event Information by the Japan Council for Quality Health Care is to share useful information for medical safety measures among a wide range of medical institutions by analyzing and providing accident information from medical institutions nationwide and to further promote medical safety measures. The project was initiated in 2004, and in 2022, there were 1158 participating facilities and 5315 reported cases [7].

Data collection

Data were collected from the database of the Japan Council for Quality Health Care on medical accidents, by searching for cases that included all "sedation" and "midazolam," all "sedation" and "propofol," and all "sedation" and "dexmedetomidine" by keyword entry, using the reporting case category "medical accident information" and reporting year "January 2010 to June 2022" as the search criteria. Duplicate cases were excluded from the retrieved cases, and after careful examination of the case details, only cases of sedation for procedural purposes were included in the study. Accidents related to intubation, postoperative sedation, procedures unrelated to sedation (e.g., erroneous tooth extraction or perforation), and cases in which the data did not allow factor analysis were excluded. A total of 133 cases were included in this analysis. The maximum number of data available at this time was 133 cases. We emphasized that the data should be widely available from all over Japan rather than from a limited number of facilities. Accident reports include two types of data: quantitative and qualitative.

Quantitative analysis

The following variables were used: year of report, patient gender, age, department, place of occurrence, nature of accident, drugs used, outcome, and establishment of an accident investigation committee.

Qualitative analysis

The accident was reviewed and analyzed using content analysis, based on an overview of the background factors, remedial measures, etiological factors, and people involved. Content analysis is useful for identifying trends and patterns in documents [8,9]. The factors of the accident were classified according to the factors of the P-mSHELL model. The classification was done individually, and the results of the classification were reviewed for any objections to the classification results, which were unanimous. The seven factors that make up the P-mSHELL model are P (patient: constitution, sudden changes caused by the patient), m (management: organizational, administrative, and systemic issues), S (software: manuals, training forms, etc), H (hardware: medical equipment, instruments, facilities, etc.), E (environment: environment affecting work and behavior), L (liveware: human error (lack of attention, errors in judgment, errors in confirmation, lack of knowledge and skill, medication errors)). The L factor includes L1 (liveware: party concerned) and L2 (other liveware: related party) factors. Originally, the L factors in the P-mSHELL model were distinguished into L1 and L2 factors. However, in this study, L1 and L2 factors were unified as L factors because of the difficulty in classifying L1 and L2 from the data. This modified model used will be hereafter described as the P-mSHEL model.

Statistical analysis

Logistic regression analysis was performed with and without each outcome factor (residual disability or not) and P-mSHEL factor. In addition, cross-tabulations were performed for the outcome (residual disability or not) and the presence of each P-mSHEL factor, time of the accident (preoperative, intraoperative, or postoperative), and drug used (propofol, midazolam, or flumazenil). Fisher's exact probability test or Chi-square test, as appropriate, and residual analysis were performed for each factor. The significance level was set at less than 5%. For the outcome (residual disability or not), three items (no disability, no probability of residual disability, and low probability of residual disability) were defined as "no residual disability", while two items (high probability of residual disability and death) were defined as "with residual disability". Fisher's exact probability test, Chi-square test, and residual analysis were used to determine the relationship between cases of respiratory depression, hypoxia, and airway obstruction, and whether the drug was a single drug (sedative only or sedative and antagonist) or multiple drug combination (sedative, analgesic, or other combination). The significance level was set at less than 5%. IBM SPSS Statistics for Windows, version 28.0 (IBM Corp., Armonk, NY, USA) was used for the statistical analysis.

## Results

The characteristics of the cases are summarized in Tables 1, 2. The number of reported accidents was on the rise, with male and female cases being 64.7% and 35.3%, respectively. Most patients were in their 60s or older. Although the majority of accidents occurred at sedation sites, approximately 30% of accidents occurred outside of sedation sites. Midazolam was the main used drug. The combined percentage of "no disability," "no possibility of residual disability," and "low possibility of residual disability" for the outcome was 85.6%. Regarding the P-mSHEL factors, L factors were present in all cases, and S factors were also common. In most cases, an accident investigation committee was established, and the largest number of cases were reviewed by an existing committee on medical safety.

**Table 1:** Sedation accidents overview Percentages are rounded to two decimal places.

Accident reporting year	n=133, n(%)	Gender	n=133, n(%)	Age	n=133, n(%)	Department (with duplicates)	n=133	Occurrence Location	n=133
2010	0(0)	male	86(64.7)	0	12(9.0)	gastroenterology	54	sedation enforcement location	93
2011	1(0.8)	female	47(35.3)	teens	2(1.5)	gastroenterology and Metabolism	1	other than sedation enforcement location	40
2012	0(0)	-	-	20s	3(2.3)	internal Medicine	23	hospital room	30
2013	1(0.8)	-	-	30s	6(4.5)	respiratory medicine	14	ICU	2
2014	3(2.3)	-	-	40s	8(6.0)	pediatrics	13	CCU	1
2015	6(4.5)	-	-	50s	6(4.5)	general Surgery	8	endoscope Recovery Room	1
2016	8(6.0)	-	-	60s	25(18.8)	orthopedic Surgery	6	MRI Preparation Room	1
2017	23(17.3)	-	-	70s	42(31.6)	cardiology	6	stairs	2
2018	15(11.3)	-	-	80s	23(17.3)	anesthesiology	4	corridor	1
2019	22(16.5)	-	-	90s	6(4.5)	emergency department	4	unknown	2
2020	19(14.3)	-	-	-	-	otorhinolaryngology	3	-	-
2021	29(21.8)	-	-	-	-	endoscopy department	3	-	-
2022	6(4.5)	-	-	-	-	dermatology	2	-	-
-	-	-	-	-	-	radiology	2	-	-
-	-	-	-	-	-	urology	2	-	-
-	-	-	-	-	-	ophthalmology	2	-	-
-	-	-	-	-	-	dental surgery	2	-	-
-	-	-	-	-	-	dentistry	2	-	-
-	-	-	-	-	-	hematology	2	-	-
-	-	-	-	-	-	neurology	2	-	-
-	-	-	-	-	-	other departments	13	-	-

**Table 2:** Sedation accidents overview Percentages are rounded to two decimal places.

Drugs used (with duplicates)	n=133	Outcome	n=133, n(%)	P-mSHEL factor (with duplicates)	n=133, n (%)	Establishment of Accident Investigation Committee	n=133, n (%)
Midazolam	115	no disability	41(30.8)	P factor	15(11.2)	existing committees on medical safety	112(84.2)
Flumazenil	42	no probability of residual disability	41(30.8)	m factor	82(61.7)	establishment of internal accident investigation Committee	19(14.3)
Pentazine	25	low probability of residual disability	32(24.0)	S factor	132(98.5)	establishment of external accident investigation committee	1(0.8)
Propofol	24	high probability of residual disability	12(9.0)	H factor	6(4.5)	in-hospital medical accident conference	1(0.8)
Pethidine	17	death	6(4.5)	E factor	2(1.5)	hearing from the department concerned	1(0.8)
Fentanyl	6	unknown	1(0.8)	L factor	133(100)	not held / None	3(2.3)
Thiopental	4	-	-	-	-	blank/unknown	3(2.3)
Trichloro-syrup	4	-	-	-	-	undecided	4(3.0)
Dexmedetomidine	4	-	-	-	-	-	-
Hydroxyzine	3	-	-	-	-	-	-
Ketamine	3	-	-	-	-	-	-
atropine	2	-	-	-	-	-	-
Butylscopolamine	1	-	-	-	-	-	-
Haloperidol	1	-	-	-	-	-	-
Thiamylal	1	-	-	-	-	-	-
Diazepam	1	-	-	-	-	-	-

Regarding the influence of P-mSHEL factors on the outcome (residual disability or not), the P factor (odds ratio = 6.347, 95% confidence interval = 2.000, 20.144) was a factor for having a residual disability (Table 3).

**Table 3:** Results of logistic regression analysis of P-mSHEL factors affecting outcome OR: odds ratio; CI: confidence interval *p<0.05

P-mSHEL factor	With residual disability (high probability of residual disability/death)	
	OR (95 % CI)	*p* value
P factor	6.347 (2.000, 20.144)	0.002*
m factor	2.193 (0.644, 7.462)	0.209
S factor	0.178 (0.007, 4.649)	0.3
H factor	0.960 (0.087, 10.581)	0.973
E factor	0 (0, -)	0.999
L factor	1.627 (0.028, 93.168)	0.814

The outcome (residual disability or not) and presence or absence of each P-mSHEL factor were significantly and differentially associated with the presence of the P factor (*p* =0.041) (Table 4).

**Table 4:** Relationship between P-mSHEL factors and outcome P-mSHEL: Patients, management, software, hardware, environment, and liveware * Not subject to approval; ^a^ Fisher’s exact test; ^b^ χ^2^ test

P-mSHEL factor		Number of subjects	No residual disability (no disability, no probability of residual disability, and low probability of residual disability)	With residual disability (high probability of residual disability/death)	*p* value
P factor	absence	118	104	14	0.041^a^
	presence	15	10	5	
m factor	absence	51	46	5	0.244^b^
	presence	82	68	14	
S factor	absence	1	0	1	0.143^a^
	presence	132	114	18	
H factor	absence	127	109	18	1^a^
	presence	6	5	1	
E factor	absence	131	112	19	1^a^
	presence	2	2	0	
L factor	absence	0	0	0	*
	presence	133	114	19	

There was a significant association between outcome (residual disability or not) and time of the accident (preoperative, intraoperative, and postoperative) (*p* =0.01), and residual analysis showed that there were significantly fewer intraoperative "with residual disability” cases and postoperative "no residual disability" cases, while postoperative "with residual disability" and intraoperative "no residual disability" cases were significantly more common. For the definition of postoperative, the start was defined as immediately after the surgery/treatment, and no time was set for the end. As a result, no cases had accidents after more than 24 hours (Table 5).

**Table 5:** Relationship between time of accident and outcome Using χ^2^ test; Residual analysis *less **more.

Time of accident		Number of subjects	No residual disability (no disability, no probability of residual disability, and low probability of residual disability)	With residual disability (high probability of residual disability/death)	*p* value
Preoperative		7	7	0	0.01
Intraoperative		87	**79	*8	
Postoperative		39	*28	**11	

There was no significant association between the outcome (residual disability) and the drug used (propofol, *p* =1; midazolam, *p* =0.722; and flumazenil, *p* =0.594) (Table 6).

**Table 6:** Relationship between drugs used and outcome ^a^ Fisher's exact test; ^b^ χ^2^ test

Drugs used		Number of subjects	No residual disability (no disability, no probability of residual disability, and low probability of residual disability)	With residual disability (high probability of residual disability/death)	*p* value
Propofol	not used	107	92	15	1^a^
	used	26	22	4	
Midazolam	not used	18	3	15	0.722^a^
	used	115	99	16	
Flumazenil	not used	91	77	14	0.594^b^
	used	42	37	5	

Respiratory depression was the most common type of accident throughout the perioperative period (Table 7).

**Table 7:** Accident Summary ENBD: Endoscopic nasobiliary drainage; PICC: Peripherally inserted central venous catheter

Time of Occurrence	Number of accidents (%)	Case Details	Number of accidents (%)
Preoperative	7(5.3)	Respiratory depression	6(4.5)
		Contraindicated drug administration (anaphylactic shock)	1(0.8)
Intraoperative	87(65.4)	Respiratory depression	31(23.3)
		Cardiac arrest	20(15.0)
		Drug dosage error	15(11.3)
		Wrong drug administered	9(6.8)
		Use of another patient's syringe	2(1.5)
		Contraindicated Drug Administration	2(1.5)
		Fall in blood pressure	3(2.3)
		Other (falls, tooth loss, severe body movement, foreign body, oversedation (details unknown))	5(3.8)
Postoperative	39(29.3)	Falling down	15(11.3)
		Cardiac arrest	7(5.3)
		Respiratory depression/hypoxia	5(3.8)
		Delayed and defective awakening	4(3.0)
		Bruising and dislocation during positional changes	2(1.5)
		Others (tachycardia, cerebral infarction, brachial plexus disorder, ENBD tube self-removal, PICC dislodgement, tooth loss)	6(4.5)

There was a significant association between instances of respiratory depression, hypoxia, and airway obstruction and whether the drug was a single agent (sedative only or sedative and antagonist) or multiple agents (sedative and analgesic or other combinations) (*p* <0.001) (Table 8).

**Table 8:** Relationship between drugs and respiratory suppression using χ^2^ test; residual analysis *less **more

Drugs	Number of subjects	Respiratory suppression		p value
		absence	presence	
Single drug	75	**54	*21	<0.001
Multi-drug combination	58	*23	**35	

Table 9 shows the percentage of each P-mSHELL factor present during sedation accidents. The L factor was present in all cases, and the S factor was in 98.5% of cases. The P factor was present in fewer cases (11.2%), the H factor in 4.5%, and the E factor in even fewer cases (1.5%). m factor was present in 61.7% of cases.

**Table 9:** Percentage of P-mSHEL factors in sedation accidents

P-mSHEL factor	Contents	Percentage of accidents (%)
P	patient: constitution, sudden changes caused by the patient	11.2
m	management: organizational, administrative, and systemic issues	61.7
S	software: manuals, training forms, etc	98.5
H	hardware: medical equipment, instruments, facilities, etc.	4.5
E	environment: environment affecting work and behavior	1.5
L	liveware: human error (lack of attention, errors in judgment, errors in confirmation, lack of knowledge and skill, medication errors)	100

For remedial measures, we identified those considered relevant to the L factor (Table 10).

**Table 10:** Countermeasures for L factors CO_2_: Carbon dioxide; ECG: Electrocardiogram; BIS: Bispectral index

Type of Measures	n (%)
-	219(100.0)
Drug-related (proper selection, proper dosage, double checking, knowledge, etc.)	52(23.7)
Thorough monitoring and recording (CO_2_ monitoring, ECG, BIS, respiratory heart rate monitoring, transport monitoring)	22(10.0)
Preoperative risk assessment	22(10.0)
Staff collaboration and training	17(7.8)
Manual creation, revision, and rule compliance	14(6.4)
Evaluation upon awakening, recovery management	14(6.4)
Anesthesiology consulting and collaboration with physicians in other departments	8(3.7)
Explanation to patients	7(3.2)
Emergency response (carts, training, etc.)	5(2.3)
Review of management methods (airway, positioning, restraint methods)	4(1.8)

## Discussion

This study classified medical accidents during sedation in Japan and analyzed their causes using the P-mSHELL model. During the study period, the number of medical accident reports increased in the second half of the year, before 2016 and after 2017. The Medical Accident Information Collection Project was initiated in 2004. The overall number of medical accidents reported in the project increased from 1,265 in 2005 to 2,703 in 2010, 3,882 in 2016, 4,095 in 2017, and 5,243 in 2021.

Most cases of sedation accidents in this study occurred in medical departments, with only a few occurring in dentistry. This does not necessarily mean that there are fewer incidents of sedation accidents in dentistry, but that there are fewer reported cases. A previous study [8] has also stated that of the 6,071 medical incidents reported from January 2014 to January 2019, 5,927 were from physicians and 144 were from dentists. At the same time, the number of incidents may be lower in dentistry because many sedation cases are undertaken by dental anesthesiologists, whereas in medicine, sedation is often undertaken by non-specialized physicians.

In the medical departments, the highest number of cases were related to gastroenterology (55 cases). These incidents are most common during endoscopic retrograde cholangiopancreatography and other endoscopic procedures. Recently, the demand for sedation has been increasing not only for endoscopic procedures but also for endoscopy [10]. The Japanese Society of Gastrointestinal Endoscopy, in cooperation with the Japanese Society of Anesthesiology, published "Guidelines for Sedation in Endoscopic Procedures" in 2013 and its second edition in 2020. Few drugs have been approved for insurance coverage for sedation during endoscopic use [11], and midazolam will be approved for off-label use for sedation in gastrointestinal endoscopy in 2023. This differs from the situation in dentistry and oral surgery, where midazolam is approved for sedation during surgery and procedures.

Accidents occurred mainly where sedatives were administered, however, they also occurred in other locations, especially in hospital rooms. Since sedation in hospital rooms was excluded, all accidents in this study occurred after the patient returned to the room. Reduced incidence of sedation accidents may be achieved by monitoring recovery after the end of sedation. On the other hand, an analysis of court cases by the American Society of Anesthesiologists found that most sedation-related deaths occurred when sedation was performed outside the operating room [12,13]. The risk of accidents increases when sedation is performed in areas with inadequate monitoring and emergency response equipment. This result means that any sedation case should be performed in a well-equipped facility.

Respiratory depression was the main type of accident in this study. In an analysis of court cases by the American Society of Anesthesiologists, respiratory depression was similarly the main cause of sedation-related deaths [12,13]. Deep sedation can induce loss of consciousness and defensive reflexes, leading to respiratory depression and pulmonary aspiration. Hence, in addition to the patient's general condition, the assessment of the airway is very important [14]. Furthermore, a large prospective study by Koers et al. reported that most adverse events during deep sedation were related to breathing and airways [15]. Sedation in dentistry and oral surgery is considered to have a high risk of upper airway obstruction due to the overlap of the airway with the operative field, as well as head retroversion and opening [16]. Some reports [2] indicate that this poses a higher risk for pediatric patients. In this study, the level of sedation could not be assessed from the data.

There were no significant differences in outcomes when comparing the type of drug used alone. ASA-SED recommends that antagonists should be promptly administered in cases of benzodiazepine or opioid administration [1]. On the other hand, the use of flumazenil after conscious sedation with midazolam in the United Kingdom was reported by The National Patient Safety Agency in 2008, which recommended against routine reliance on flumazenil for sedation relief and encouraged regular audits to identify midazolam overdose problems. In fact, flumazenil is used less frequently [17]. In this study, there was no difference in outcome between the use of midazolam alone and in combination with flumazenil.

Respiratory depression, hypoxia, and airway obstruction were observed regardless of whether the drug was a single agent (sedative only, sedative, or antagonist) or multiple agents (sedative, analgesic, or other combination); however, were more often observed with the use of multiple agents. The concomitant use of opioids and benzodiazepines has been reported to contribute to morbidity and mortality from sedation in gastrointestinal endoscopy [18]. The combination of drugs increases unpredictability and decreases safety margins compared to single-agent procedures. Therefore, the risk of adverse events may increase, and the risk of respiratory depression associated with the combination of fentanyl and central nervous system depressants, such as benzodiazepines, is high. The risk of a strong drug interaction between fentanyl and other opioids in combination with midazolam and the increased risk of hypoxemia and apnea has long been noted [19]. However, it has also been reported that the combination of midazolam and fentanyl in dental treatment for dental phobia is safe and effective for transient respiratory depression, but no other physiological changes were detected [20]. In general, low doses of midazolam have little effect on the respiratory system when administered for moderate sedation. However, it can cause respiratory depression and apnea in a dose-dependent manner or when used with fentanyl or propofol. As the required depth of sedation varies with the type and invasiveness of the procedure, sedation in different departments cannot be compared. However, a comparison of sedation in several departments in this study showed that respiratory depression was often seen with multiple drug use, and special care should be taken when using multiple drugs. For example, sedation by a physician or dentist specializing in anesthesia with sufficient drug knowledge and clinical experience is applicable.

No disability, no probability of residual disability, and low possibility of residual disability accounted for more than 80% of the outcomes in this study. There was a statistically significant difference in the relationship between the timing of the accident and the presence or absence of residual disability, with accidents that occurred in the postoperative period having more residual disability. The severity of the postoperative outcome suggests that careful monitoring is warranted after the completion of sedation. In doing so, the half-life of the antagonist, in particular, must also be fully considered.

In this study, all cases had an L factor, and 98.5% of the cases also had an S factor. The m factor was present in 61.2% of cases; however, the P factor was present in fewer cases (11.2%), as were the H and E factors. Surprisingly, among the factors related to residual disability, only the P factor influenced the presence of residual disability. In this regard, a preoperative risk assessment of the patient's general condition and airway is extremely important. According to Koers et al. [15], the rate of adverse events increases with ASA physical status for deep sedation in patients with ASA physical status of 3 and 4. Proper risk assessment of the patient prior to sedation (to the same extent as that done during general anesthesia), appropriate intraoperative and postoperative monitoring, and preparation for emergency response contribute to accident prevention.

S factor relates to manuals, education, and training styles; m factor relates to organizational, administrative, and corrective issues; E factor relates to the environment that affects work and behavior; and H factor relates to hardware, such as medical equipment, instruments, facilities, and facility structure. Improving human factors in anesthesia involves a variety of elements, including medical equipment design, procurement processes, pharmaceutical design, operating room design and layout, hospital design, scheduling and staffing related to surgery, checklists, protocols, teamwork, communication, education, and training [21]. In other words, S, H, E, and m factors are closely related to L factors as potential factors that induce human error. Therefore, it can be said that the S factor, which is related to manuals and education and training formats, resulted in almost the same percentage of accidents as the L factor in this study, and the m factor also contributed a high percentage. One possible reason for the same percentage of S and L factors could be the influence of the L factor on the S factor since it is the medical professionals who design and enforce the manuals, and education and training. Properly designed checklists, when used as intended, have been shown to improve protocol compliance [22], teamwork, and communication [23]. The main countermeasures for the L factor are drug-related suggestions, followed by thorough monitoring, recording, and preoperative risk assessment. Human error is said to tend to be overlooked [8,24]. In this study, staff collaboration, education, and cooperation with anesthesiology consultants and physicians in other departments were not high on the list of measures to improve the L factor. However, drug-related suggestions specifically include improving drug-related knowledge and the associated appropriate selection of drug types and doses, and double-checking when administering drugs. The content is expected to include both increased awareness among individual medical professionals and collaboration among staff members. Various monitors are H-factor measures, whereas drug handling and preoperative risk assessment methods are S-factor measures. However, human beings make the final decision to use a monitor or drug. In this respect, many remedial measures for the L factor have been mentioned, and it can be said that human error was also considered.

The number of H and E factors detected in this study was small. This suggests that the current medical environment, facilities, and equipment are standard. However, as new monitoring equipment will be introduced in the future, safety is expected to improve. In addition to this, Factor H is a factor that is easily overlooked from reporting unless there is an obvious malfunction or failure of equipment, instruments, or facilities; Factor E is also easily overlooked unless there is an obvious problem with lighting, smell, or sound.

This study only covered the number of cases and items reported. Its limitations include, first, that because it is a secondary analysis of data, the analysis is limited to statements made by the reporter, and each factor in the P-mSHEL is a potentially confounding factor. Furthermore, because the analysis is based on the reporter's description, the patient's risk assessment is ambiguous, and the details of the healthcare provider's situation and interventions are unknown. Therefore, it could not be ascertained whether the person administering sedation was an anesthesiologist or a non-anesthesiologist. Also, the number of data is 133 cases at this time, but more valid analysis will be possible as more cases are reported in the future. Future studies will need to examine the impact of specific P factor characteristics, the effectiveness of different interventions to improve the L factors, or the impact of the introduction of new monitoring techniques.

## Conclusions

The analysis in this study is based on reports of sedation accidents in Japan. It has been found that despite the existence of many guidelines, accidents continue to occur. Based on the P-mSHEL model used, the factors contributing to accidents were mostly L and S factors, with L factors being found in all cases. However, only P factors affected outcome severity. The main accident was respiratory depression. Respiratory depression was observed in many patients, particularly with the use of multiple drugs, and more serious disabilities were found in accidents that occurred postoperatively. Since multiple drug use increases the risk of respiratory depression and should be performed under particularly tight supervision by anesthesiologists. Non-anesthesiologists may be better off using single agents. Strict adherence to guidelines and thorough perioperative monitoring are important to prevent sedation accidents. Furthermore, it is especially important to know the patient's condition in advance, and strict postoperative management is also required to prevent serious accidents, especially in high-risk patients.
